# Editorial: Lipid-immune interplay in chronic inflammatory-based disorders

**DOI:** 10.3389/fimmu.2026.2016456

**Published:** 2026-10-19

**Authors:** Paola De Cicco, Undurti N. Das, Maria Francesca Nanì, Barbara Romano

**Affiliations:** 1 Department of Pharmacy, School of Medicine and Surgery, University of Naples Federico II, Naples, Italy; 2 UND Life Sciences LLC, Battle Ground, WA, United States

**Keywords:** abnormal sphingolipid metabolism, acipimox, AGE-RAGE pathways, alanine aminotransferase, AMPK, AMPK activators, apoE-deficient mice, apoptosis

Lipid metabolism and immune regulation intersect across chronic inflammatory disorders, providing opportunities to understand how metabolic disturbances accompany, sustain or modify tissue injury. The 17 contributions to this Research Topic examine these relationships through experimental studies, clinical association analyses, genetic approaches and reviews. Together, they connect hepatic and systemic lipid abnormalities with inflammatory signaling, immune-cell phenotypes and organ-specific pathology. Their diversity also calls for attention to the strength and scope of the evidence: molecular mechanisms established in experimental models, genetically inferred relationships and clinical associations contribute different insights. A central message is that therapeutic investigation should consider lipid metabolism and immune regulation together, while preserving the biological context in which each relationship is observed.

Metabolic liver disease provides a prominent setting for this interplay. Hines et al. investigated TNF receptor signaling in mice fed a choline-deficient diet and in isolated hepatocytes. Compared with wild-type mice, TNFR1-deficient animals showed less hepatic lipid accumulation and injury, higher lipid oxidation and increased hepatic SIRT1 expression. In hepatocytes, TNF suppressed Sirt1 expression while inducing DBC1, an inhibitor of SIRT1. These findings support a role for TNFα/TNFR1 signaling in hepatic lipid metabolism and connect inflammatory signaling with changes in a metabolic regulator. Complementary reviews broaden this perspective. Feng et al. discuss AMPK in metabolic dysfunction-associated steatotic liver disease (MASLD), covering lipid metabolism, inflammation, apoptosis and fibrosis, alongside the development and translational challenges of AMPK activators. Shi et al. examine TREM2-positive macrophages in metabolic dysfunction-associated steatohepatitis (MASH), emphasizing their involvement in lipid handling and immune regulation. These contributions identify hepatocyte metabolism and macrophage responses as related areas for mechanistic and therapeutic investigation.

The study by Liu et al. on Huatan Qushi Huoxue formula (HQHF) illustrates an approach combining experimental assessment with computational target prioritization. In a high-fat diet-induced mouse model of metabolic associated fatty liver disease (MAFLD), HQHF reduced steatosis, inflammatory-cell infiltration, triglyceride levels and markers of liver injury and oxidative stress. Network pharmacology identified candidate targets enriched in MAPK, Toll-like receptor, IL-17 and AGE-RAGE pathways, while experimental analyses showed downregulation of selected inflammatory mediators and proteins. The findings support concurrent lipid-lowering and anti-inflammatory effects in this model, with the computational analyses proposing pathways for further investigation. The distinction between predicted pathway involvement and experimentally measured responses is particularly relevant when interpreting the mechanisms of multicomponent interventions.

Two clinical studies extend the liver-focused discussion beyond experimental mechanisms. In a retrospective case-control study involving 522 propensity score-matched participants, Liang et al. found that the neutrophil-to-HDL cholesterol ratio (NHR) and systemic inflammation response index (SIRI) were independently associated with MASLD. Combining these indices with alanine aminotransferase improved discrimination compared with alanine aminotransferase alone, with an area under the curve of 0.865 and internal bootstrap validation. These results support further evaluation of accessible composite markers for identifying MASLD, although prospective predictive performance remains to be established. In their study of dyslipidemia and chronic liver disease, Li et al. analyzed 6,926 CHARLS participants and reported an association in both analytical directions. Depressive symptoms statistically mediated a small proportion of these associations, accounting for 2.91% and 2.54%, respectively. The cross-sectional design precludes causal inference, but the study adds a psychosocial dimension to the clinical relationship between lipid abnormalities and liver disease.

Cardiovascular and autoimmune contributions address additional connections between lipid disturbances and inflammatory regulation. Cong et al. propose the SIRT1/STAT3 axis as an integrating framework in rheumatoid arthritis. Their review describes an antagonistic relationship in which SIRT1 deacetylates and inhibits STAT3, linking metabolic sensing with Th17 differentiation, synovial pathology and dyslipidemia. It also discusses SIRT1 activators, STAT3 inhibitors and dietary interventions as potential strategies to address inflammatory and metabolic abnormalities. In atherosclerosis, Zhang et al. integrated gene-expression analyses, expression quantitative trait loci and Mendelian randomization, implicating CLEC5A and ISG20 in disease susceptibility. The experimental results described for ISG20 showed increased expression in oxidized LDL-stimulated macrophages and ApoE-deficient mice, with localization to endothelial cell- and macrophage-rich regions of plaques. These findings prioritize candidates for further functional investigation and connect genetic evidence with disease-associated expression patterns.

A complementary pharmacological contribution comes from Liu et al. in their study of gemilukast. This cysteinyl leukotriene receptor antagonist reduced cholesterol uptake in Caco-2 cells and cholesterol solubility in mixed micelles. In LPS-stimulated macrophages, it decreased pro-inflammatory cytokines and promoted M1-to-M2 repolarization, accompanied by reduced PI3K/AKT phosphorylation. Lipid-lowering effects were also observed in rat and mouse models, with reduced circulating inflammatory cytokines in high-fat diet-fed mice. These preclinical findings provide a rationale for investigating agents with combined effects on cholesterol handling and inflammatory responses. They also illustrate how cellular and whole-animal experiments can contribute complementary evidence when evaluating an immunometabolic pharmacological profile.

The importance of cellular context is particularly apparent in inflammatory skin disease. Masuda-Kuroki et al. investigated sphingosine-1-phosphate receptor 2 (S1PR2) in mouse models of imiquimod-induced psoriasis-like inflammation. Keratinocyte-specific S1pr2 deletion worsened inflammatory responses and increased Th17-cell populations in skin-draining lymph nodes, supporting a restraining role for keratinocyte S1PR2 signaling. The study suggests that stimulating this receptor warrants investigation as a potential therapeutic approach. In rosacea-like dermatitis, Xu et al. identified lithospermic acid as a candidate KLK5 inhibitor and reported improvement in an LL-37-induced mouse model, together with inhibition of KLK5 and TLR4/NF-κB-related inflammation. Metabolomic analysis highlighted correction of metabolic disturbances, particularly phenylalanine metabolism. The rosacea study therefore broadens the collection’s immunometabolic scope, whereas the S1PR2 study directly illustrates how lipid sensing in epithelial cells can shape inflammatory responses.

Genetic and review-based contributions further develop the relationship between lipid abnormalities and organ injury. Xiao et al. used Mendelian randomization to investigate lipid species and ulcerative colitis, followed by mediation analyses of immune-cell phenotypes. The analyses supported potential causal relationships for six genetically predicted lipid species and implicated specific myeloid-cell phenotypes as mediators. In particular, the phenotype described as CD11b on CD33+ HLA-DR+ CD14dim mediated part of the relationship between PC and ulcerative colitis, with a reported mediation proportion of 15.38%. This finding motivates investigation of lipid-myeloid relationships without establishing a tissue-localized cellular mechanism. In diabetic kidney disease, Shang et al. review evidence linking podocyte injury to lipid peroxidation, abnormal sphingolipid metabolism and cholesterol accumulation, together with the regulatory roles of SREBP1, PPARα and the NLRP3 inflammasome. The review by Krishnan et al. adds a resolution-focused perspective by discussing regulatory T cells and resolvins, including their potential implications for insulin resistance, obesity and type 2 diabetes. These articles connect lipid-related processes with immune regulation and tissue dysfunction at distinct levels of analysis.

The collection also includes an acute injury setting with potential relevance to subsequent neurological complications. O’Leary et al. examined prefrontal cortical lipid profiles in rats following severe burns. Lipidomics identified changes in selected lipid classes, and acipimox treatment reduced lipid accumulation, with reported restoration to sham levels. Changes in inflammatory gene expression and lipid-inflammatory marker correlations accompanied these findings. This work connects peripheral injury with altered cerebral lipid homeostasis and suggests that inhibiting lipolysis may modify these responses. The results provide an experimental basis for further investigation of the relationship between systemic metabolic changes and neuroinflammation; their implications for cognitive outcomes and clinical treatment require additional study.

At the cellular level, Schöl et al. investigated the protein environment of neutral sphingomyelinase-2 (nSMase2) in TNFα-stimulated Jurkat cells. Using optimized proximity labeling and quantitative mass spectrometry, they identified rapid changes in the nSMase2-proximal protein network within the first five minutes of stimulation. The proximitome of enzymatically active nSMase2 became enriched in proteins associated with vesicle-mediated transport, including recycling endosomes, the trans-Golgi network and exocytic vesicles. These data provide a resource for examining how sphingolipid metabolism relates to early cytokine signaling and intracellular trafficking. They also add a spatial dimension to the collection by focusing on dynamic protein networks surrounding a lipid-metabolizing enzyme.

The hypothesis and theory article by Maddipati offers a distinct conceptual perspective through “unalamation,” defined as the physiological activity of inflammatory mediators maintained in balance with anti-inflammatory mediators without clinical inflammation. The article proposes that disturbance of this balance may contribute to chronic inflammation independently of unresolved acute injury. It further distinguishes the proposed implications for degenerative disease from those for cancer: stimulating endogenous anti-inflammatory mediators is proposed for the former, whereas local inhibition of their biosynthesis in tumors is hypothesized to favor acute inflammation and antitumor immune responses. These proposals invite discussion of mediator balance and disease context, while remaining conceptual hypotheses rather than established therapeutic principles. Their inclusion cautions against assuming that enhancing anti-inflammatory activity has equivalent consequences across all pathological settings.

Several priorities emerge from this collection. Integrating lipid measurements with immune phenotyping may help identify which metabolic changes accompany particular cellular responses. Experimental perturbation can then test the functional significance of candidates emerging from genetic analyses, omics and network pharmacology. Clinical composite indices warrant evaluation in independent populations and prospective settings, while pharmacological findings require further assessment of efficacy, selectivity and translatability. The contributions collectively support a research agenda in which lipid metabolism and immune regulation are studied as connected processes, with explicit attention to cell type, tissue and disease setting. Progress will depend on translating these connections into testable mechanisms and interventions whose benefits are established within the contexts for which they are intended.

